# Intensifying Continuous Production of Gag-HA VLPs at High Cell Density Using Stable Insect Cells Adapted to Low Culture Temperature

**DOI:** 10.3389/fbioe.2022.917746

**Published:** 2022-06-29

**Authors:** Bárbara Fernandes, Ricardo Correia, Paula M. Alves, António Roldão

**Affiliations:** ^1^ IBET-Instituto de Biologia Experimental e Tecnológica, Oeiras, Portugal; ^2^ Instituto de Tecnologia Química e Biológica António Xavier, Universidade Nova de Lisboa, Oeiras, Portugal

**Keywords:** influenza virus-like particles, insect cells, perfusion, high cell density, alternating tangential flow filtration, tangential flow filtration

## Abstract

Protein production processes based on stable insect cell lines require intensification to be competitive with the insect cell-baculovirus expression vector system (IC-BEVS). High cell density (HCD) cultures operate continuously, capable of maintaining specific production rates for extended periods of time which may lead to significant improvements in production yields. However, setting up such processes is challenging (e.g., selection of cell retention device and optimization of dilution rate), often demanding the manipulation of large volumes of culture medium with associated high cost. In this study, we developed a process for continuous production of Gag virus–like particles (VLP) pseudotyped with a model membrane protein (influenza hemagglutinin, HA) at HCD using stable insect cells adapted to low culture temperature. The impact of the cell retention device (ATF vs. TFF) and cell-specific perfusion rate (CSPR) on cell growth and protein expression kinetics was evaluated. Continuous production of Gag-HA VLPs was possible using both retention devices and CSPR of 0.04 nL/cell.d; TFF induces higher cell lysis when compared to ATF at later stages of the process (k_D_ = 0.009 vs. 0.005 h^−1^, for TFF and ATF, respectively). Reducing CSPR to 0.01–0.02 nL/cell.d using ATF had a negligible impact on specific production rates (r_HA_ = 72–68 titer/10^9^ cell.h and r_p24_ = 12–11 pg/10^6^ cell.h in all CSPR) and on particle morphology (round-shaped structures displaying HA spikes on their surface) and size distribution profile (peaks at approximately 100 nm). Notably, at these CSPRs, the amount of p24 or HA formed per volume of culture medium consumed per unit of process time increases by up to 3-fold when compared to batch and perfusion operation modes. Overall, this work demonstrates the potential of manipulating CSPRs to intensify the continuous production of Gag-HA VLPs at HCD using stable insect cells to make them an attractive alternative platform to IC-BEVS.

## 1 Introduction

Virus-like particles (VLPs) can elicit robust and broad immune responses by presenting target epitopes at their surface as repeat arrays, efficiently activating antigen-presenting cells (Fernandes et al., 2013). They are self-assembled protein complexes mimicking the conformation of a native virus but lacking the genetic information of their infectious counterparts, thus being regarded as safe vaccine candidates (Roldão et al., 2010). Numerous VLP-based vaccine candidates have been developed (Pushko et al., 2005; Krammer and Grabherr, 2010), some reaching approval and commercialization such as Engerix^®^ and Recombivax HB^®^ (hepatitis B virus), Gardasil^®^ and Cervarix^®^ (human papillomavirus), and Novavax’s vaccine against SARS-CoV-2 (Mahase, 2021). The complexity of enveloped VLPs (including influenza) demands its expression in eukaryotic systems, such as insect or mammalian cells (Nooraei et al., 2021).

The insect cell–baculovirus expression vector system (IC-BEVS) is one of the most commonly used platforms for VLP production; targeted diseases using VLP-based vaccines include HIV, influenza, chikungunya, Ebola, and dengue (Mortola and Roy, 2004; Sun et al., 2009; Krammer et al., 2010; Kuwahara and Konishi, 2010; Lynch et al., 2010; Metz et al., 2013).

Despite yielding high-protein titers in short timeframes, IC-BEVS has several limitations impairing process performance and/or product quality (e.g., release of host and viral proteases due to the lytic infection and the complexity of continuous production) (Fernandes et al., 2021). This has triggered interest in stable insect cells and the development of new bioprocess strategies for their culture aiming to improve yields (Fernandes et al., 2020; Fernandes et al., 2021).

Although batch and fed-batch processes are still preferred in the biopharmaceutical industry, continuous and perfusion processes have emerged as attractive alternatives for the production of labile proteins with special product quality requirements (Karst et al., 2016). Key hardware in scalable perfusion processes is the cell retention device. This includes spin filters, gravity settlers, acoustic filters, or hollow fibers (Voisard et al., 2003), with the latter being among the most used technology (Lin et al., 2017; Bielser et al., 2018). Filtration through hollow fibers using alternating tangential flow filtration (ATF) has become an increasingly attractive alternative to the traditional tangential flow filtration (TFF) for its claims of better culture performance and product sieving (Wang et al., 2017). The selection of the appropriate membrane material and pore size is critical to avoid membrane fouling and impacts on product retention (Nikolay et al., 2020).

As perfusion and continuous processes rely on the consumption of large volumes of media for extended periods, space-time yield (STY, protein/L.day) rather than volumetric or cell-specific yields may reflect better the overall process costs (Tapia et al., 2016). A continuous process at high cell density (HCD) capable of maintaining specific protein production rates for long periods of time can result in high volumetric titers (Konstantinov et al., 2006). However, it has become evident that the success of perfusion technology relies on the reduction of volumetric perfusion rates (Konstantinov et al., 2006). Defining the minimal cell-specific perfusion rate (CSPR) would allow for optimization of expensive media utilization, thus keeping the cost of goods low (Schulze et al., 2021).

When combined with cell-line engineering, culture medium design, and improved process control, perfusion and continuous operation modes can significantly improve the bioprocess (Schulze et al., 2021). The identification of key limiting nutrients in cell culture medium throughout production processes towards the design of personalized supplementation or the development of customized culture media formulations enriched in specific metabolites has proven to be beneficial in improving yields and/or product quality (Radhakrishnan et al., 2018; Sequeira et al., 2018).

In this study, a continuous process for Gag-HA VLP production at HCD using stable insect cells adapted to low culture temperature was developed. Cell retention devices for perfusion, long-term continuous culture, and stepwise decrease of CSPR were evaluated to determine their potential to impact cell growth and metabolism, specific protein production rate, and product quality attributes.

## 2 Materials and Methods

### 2.1 Cell Lines and Culture Media

Insect *Sf9* cells expressing Gag-HA VLPs (from now on named “Sf9 Gag-HA” cells), previously established in our lab (Fernandes et al., 2021), were routinely sub-cultured as described elsewhere (Fernandes et al., 2020). Briefly, cells were sub-cultured to 1 × 10^6^ cell/mL every 3–4 days when cell density reached 2–3 × 10^6^ cell/mL using Sf-900™ II SFM (Thermo Fisher Scientific) media.

### 2.2 Production of Gag-HA Virus-Like Particles

**FIGURE 1:**
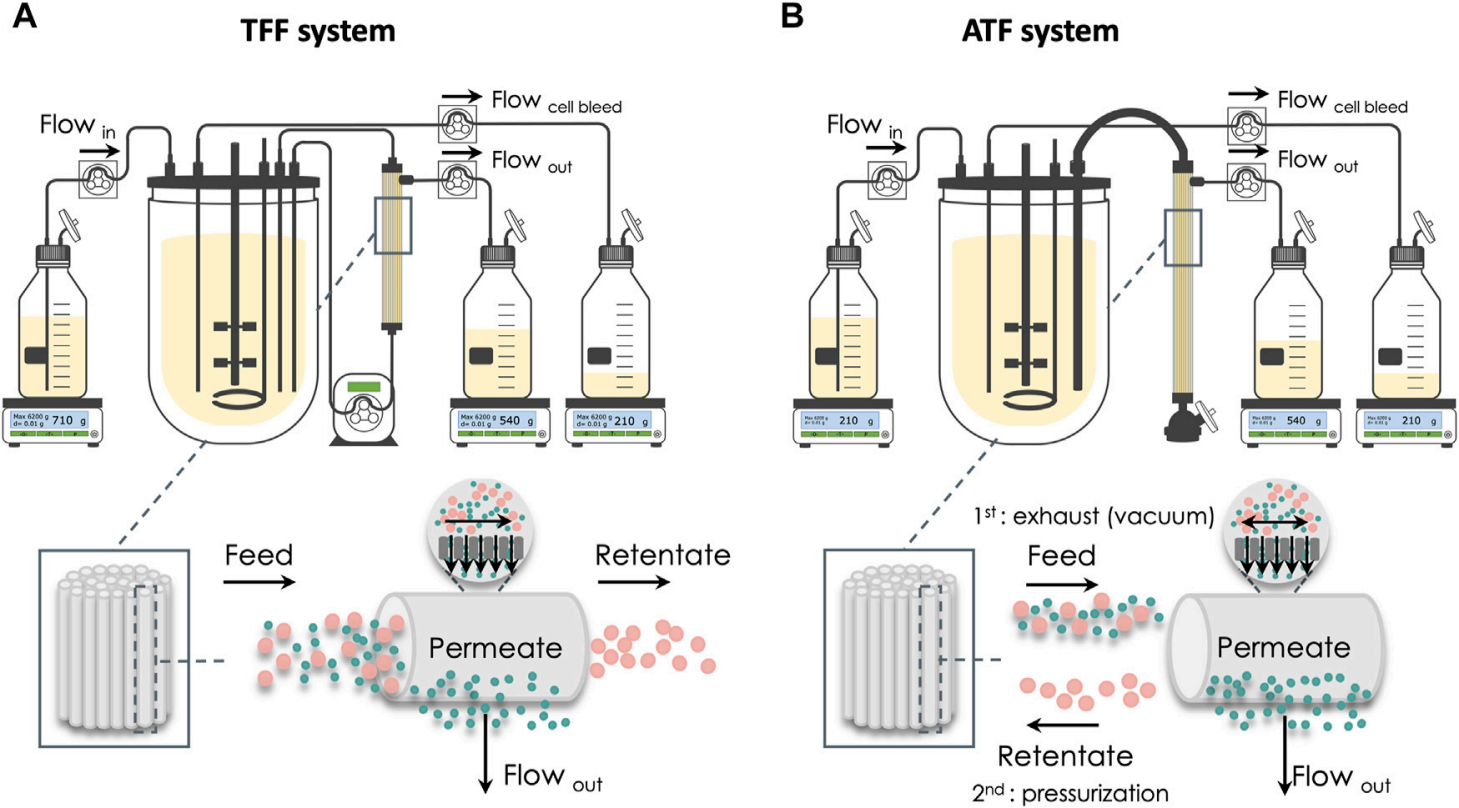
Schematic representation of the two bioreactor setups and the respective cell retention system. **(A)** Tangential flow filtration (TFF). **(B)** Alternating tangential flow filtration (ATF). In the TFF system, cell culture recirculates through the membrane surface in a single direction, with recirculation being traditionally guaranteed by using a peristaltic pump. ATF system uses a diaphragm pump that creates a bidirectional flow across the membrane.

**FIGURE 2:**
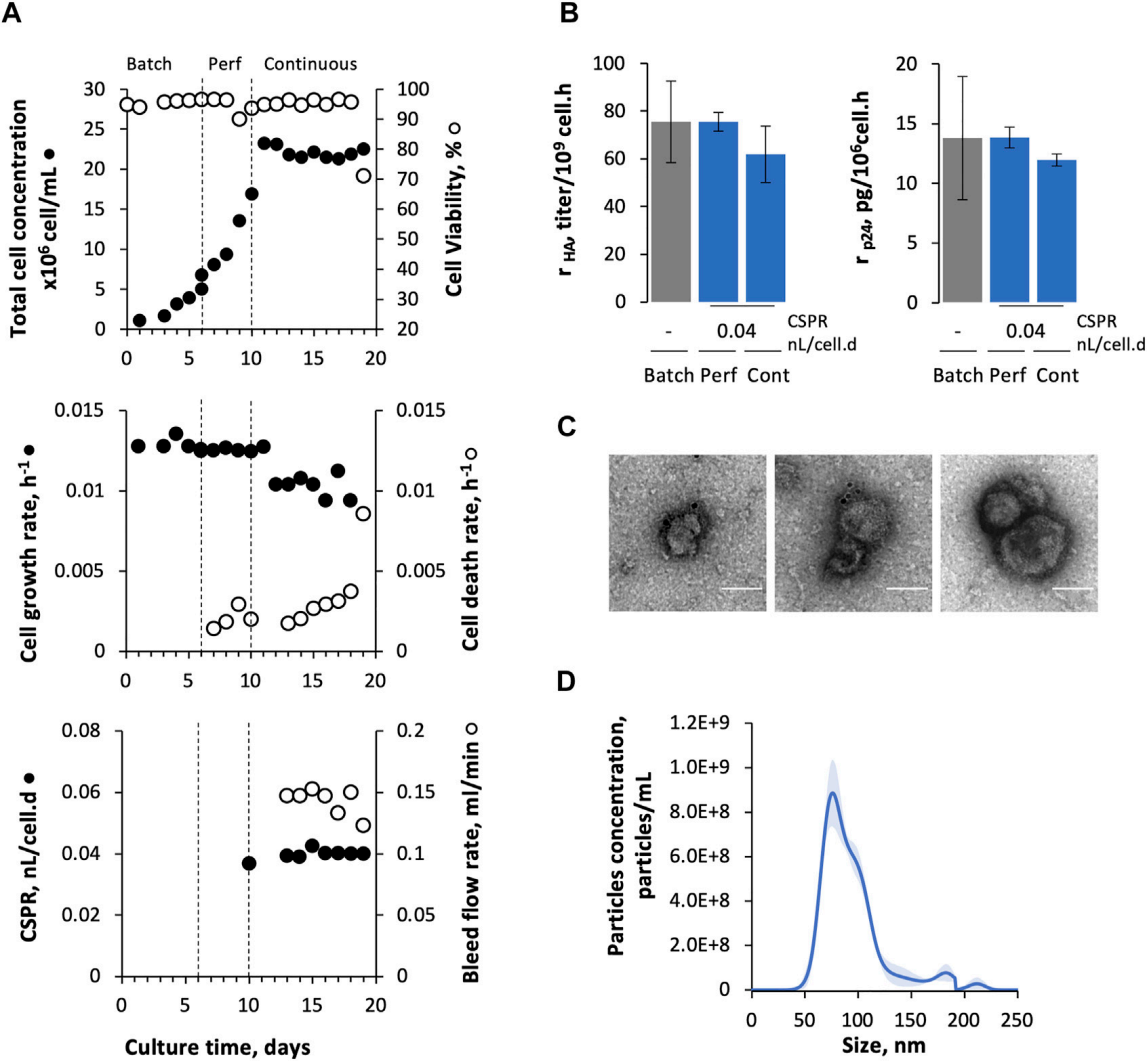
Production of Gag-HA VLPs in continuous operation mode using TFF. **(A)** Cell growth and viability kinetics, cell growth and death rate, cell-specific perfusion rate (CSPR), and bleed flow rate. **(B)** Specific HA and p24 production at different modes of operation (batch, perfusion, and continuous). **(C)** Immunogold staining TEM and **(D)** nanoparticle tracking analysis from purified and concentrated Gag-HA VLP samples. Data in bar graphs are expressed as mean ± SD of one biological replicate using data linearization. One-way ANOVA with post-hoc Tukey’s multiple comparison analysis method. The mean of each column was compared with the mean of the batch; * adjusted *p*-value < 0.05 was considered statistically significant.

The production of Gag-HA VLPs was performed in computer-controlled stirred-tank bioreactors (BIOSTAT^®^ DCU-3, Sartorius). Cells were seeded at 1 × 10^6^ cell/mL, grown up to 20 × 10^6^ cell/mL in perfusion, and then maintained at this cell concentration for up to 36 days in the continuous operation mode. Two cell retention systems were explored: 1) tangential flow filtration (TFF) using BioOptimal™ MF-SL (Asahi Kasei), a microfiltration polysulfone hollow fiber (HF) with 0.4 μm pore size, 1.4 mm lumen, and 0.005 m^2^ surface area (Figure 1A); 2) alternating tangential flow filtration (ATF) using XCell™ ATF-2 (Repligen), a microfiltration polyethersulfone HF with 0.5 μm pore size, 1 mm lumen, and 0.13 m^2^ surface area (Figure 1B). The recirculation flow was set at 0.4 and 0.6 L/min in the TFF and ATF systems, respectively. A peristaltic pump Sartoflow^®^ Slice 200 benchtop crossflow system (Sartorius) was used in the TFF system. The cell-specific perfusion rate (CSPR) was set to 0.04 nL/d.cell when glucose (Glc) and glutamine (Gln) concentrations reached 48 and 6 mmol/L, respectively (Fernandes et al., 2021). Culture volume in the bioreactor was kept constant by gravimetric feed control. pO_2_ was set to 30% of air saturation and maintained by varying the agitation rate (70–150 rpm) and the percentage of O_2_ in the gas mixture (0%–100%). The gas flow rate was set to 0.01 vvm, and the temperature was kept at 22°C. The working volume was 0.8 L.

### 2.3 Purification of Gag-HA Virus-Like Particles

Cell culture bulk was harvested and clarified by centrifugation, first at 200 g, 4°C, 10 min, for cell removal, and then at 2,000 g, 4°C, 20 min for removal of cellular debris. For purification and concentration of Gag-HA VLPs, a Sartobind Q nano 1 ml (Ref. 96IEXQ42DN-11, Sartorius) was used followed by a concentration step with a centrifugal filter unit Amicon ^®^ Ultra-15 (Ref UFC910008, Merck Millipore). The Gag-HA VLPs were stored at −80°C (long-term storage) or at 4°C (short-term storage) in HEPPES buffer (50 mM) containing NaCl (30 mM), trehalose (15% w/v) and pH 7.4.

### 2.4 Analytics

#### 2.4.1 Cell Concentration and Viability

Cell concentration and viability were assessed using the trypan blue exclusion method by using the Fuchs–Rosenthal hemocytometer chamber (Brand, Wertheim) and/or the Cedex HiRes analyzer (Roche). Cell viability was also assessed through a fluorescent membrane integrity assay to discriminate between live and dead cells. Briefly, cells were incubated with 10 μg/ml of fluorescein diacetate (FDA; Sigma-Aldrich) and 2 μg/ml of propidium iodide (PI; Sigma-Aldrich) and then observed under a fluorescence microscope (DMI6OOOB, Leica Microsystems, Wetzlar). Cells with the fluorescence metabolization product of FDA were considered alive, while cells with PI staining were considered dead, due to the presence of double-strand DNA.

#### 2.4.2 Metabolite Analysis

Glucose, glutamine, and lactate quantification were performed using the Cedex Bio analyzer 7100 (Roche). Amino acids were determined by UPLC after derivatization using the AccQ-Tag method (Waters).

#### 2.4.3 Enzyme-Linked Immunosorbent Assay (ELISA)

The concentration of p24 protein (as a proxy for Gag VLPs) was quantified using the Lenti-X p24 Rapid Titer Kit (Clontech) according to the manufacturer’s instruction and as described elsewhere (Fernandes et al., 2020).

#### 2.4.4 Hemagglutination Assay

The hemagglutination assay used herein is a plate-based assay in which the HA titer is determined by comparing the hemagglutination profile of culture samples with that of a standard of known HA concentration, and was performed as described elsewhere (Sequeira et al., 2018; Correia et al., 2020).

#### 2.4.5 Western Blot

Western blot analysis was performed as reported elsewhere (Correia et al., 2020). For HA identification, a sheep polyclonal antibody kindly provided by NIBSC (United Kingdom) was used at a dilution of 1:1000. For Gag protein identification, a mouse polyclonal antibody (Abcam, cat# ab9071) was used at a dilution of 1:1000. As a secondary antibody, an anti-mouse IgG antibody conjugated with alkaline phosphatase conjugate labeling was used at a dilution of 1:5000 (Sigma, Ref.: A3438) and an anti-sheep IgG antibody with alkaline phosphatase conjugate labeling was used at a dilution of 1:5000 (Abcam, cat# ab6901) for Gag and HA protein identification, respectively. The expected molecular weight (MW) of HA and Gag proteins are 64 and 40 kDa, respectively.

#### 2.4.6 Nanoparticle Tracking Analysis

The concentration of Gag-HA VLPs was assessed using NanoSight NS500 NTA equipment (NanoSight) as described elsewhere (Correia et al., 2020; Fernandes et al., 2020). Particles with a diameter between 100 and 200 nm were considered Gag-HA VLPs (Tretyakova et al., 2016).

#### 2.4.7 Transmission Electron Microscopy

Negative staining transmission electron microscopy (TEM) was used to assess the conformation and size of Gag-HA VLPs as described elsewhere (Fernandes et al., 2020). For identification of HA protein on the surface of Gag-VLP, immunogold labeling TEM was performed as described elsewhere (Fernandes et al., 2021).

### 2.5 Statistical Analysis

Data are presented as mean ± standard deviation. Differences were tested by one-way ANOVA with post-hoc Tukey’s multiple comparison analysis method and Dunnett’s multiple comparison test (adjusted *p*-value < 0.05 was considered statistically significant).

### 2.6 Mathematical Equations

Mathematical equations for estimation of reaction rates—cell-specific growth rate (μ), yield coefficients (Y_i,j_, mass of j formed or consumed *per* mass of i formed or consumed), specific rates of product j formed ( $r_{P_{j}}$ ) and of substrate j consumed ( $r_{{\text{S}}_{j}}$ ), cell dilution rate, CSPR, cell death rate (k_D_), and space-time yield (STY)—are provided in Supplementary Appendix S1.

### 2.7 Data Availability Statement

The sensitive nature of some of the reagents used in this study (e.g., cell lines and plasmids) means that they are only readily available internally to the author’s institution staff for R&D purposes. For external researchers, approval of reagent requests may be obtained *via* an email addressed to the corresponding author.

## 3 Results

### 3.1 Production of Gag-HA Virus-Like Particles in a Continuous Operation Mode Using Tangential Flow Filtration

Adapted Sf9 Gag-HA cells were cultured in stirred-tank bioreactors (STB) under continuous operation mode using TFF as a cell retention system (Figure 1A), and their growth and protein (p24 and HA) expression kinetics were assessed for up to 9 days (Figure 2). The cell-specific perfusion rate (CSPR) was set to 0.04 nL/cell/d.

Cells were inoculated at 1 × 10^6^ cell/mL, grown in batches for 6 days, and then in perfusion for additional 4 days until reaching 20 × 10^6^ cell/mL. Cell concentration was then maintained at approx. 20 × 10^6^ cell/mL from day 10 onwards by manipulating the bleed flow rate. The cell growth rate was constant during continuous operation mode (0.012 ± 0.001 h^−1^) and completely stopped on day 19. Indeed, cell viability dropped significantly (<70%) on day 19 and cell death rate (k_D_) (assessed by LDH quantification) reached values close to the cell growth rate reported throughout continuous mode. A fluorescent membrane integrity assay done on day 19 corroborated these results, revealing the presence of DNA filaments (as detected by PI staining) in culture samples that may be related to the cumulative cell lysis along culture time (Supplementary Figure S1A).

HA and p24 proteins could be identified by Western blot (Supplementary Figure S1B); titers were similar in bioreactor and permeate, and constant during continuous culture (Supplementary Figure S1C). Notably, specific HA and p24 production rates (r_HA_ and r_p24_, respectively) obtained in continuous mode seem to be comparable to those obtained in batch and perfusion phases (Figure 2B), and similar to previous studies (Fernandes et al., 2021). Gag-HA VLPs were purified and concentrated, and then analyzed by TEM and NTA (Figures 2C,D). TEM and NTA data confirmed the presence of particles resembling Gag VLPs (in size and morphology) with HA molecules (spikes) displayed on their surface.

### 3.2 Intensifying Continuous Gag-HA Virus-Like Particles Expression Using Alternating Tangential Flow Filtration

**FIGURE 3:**
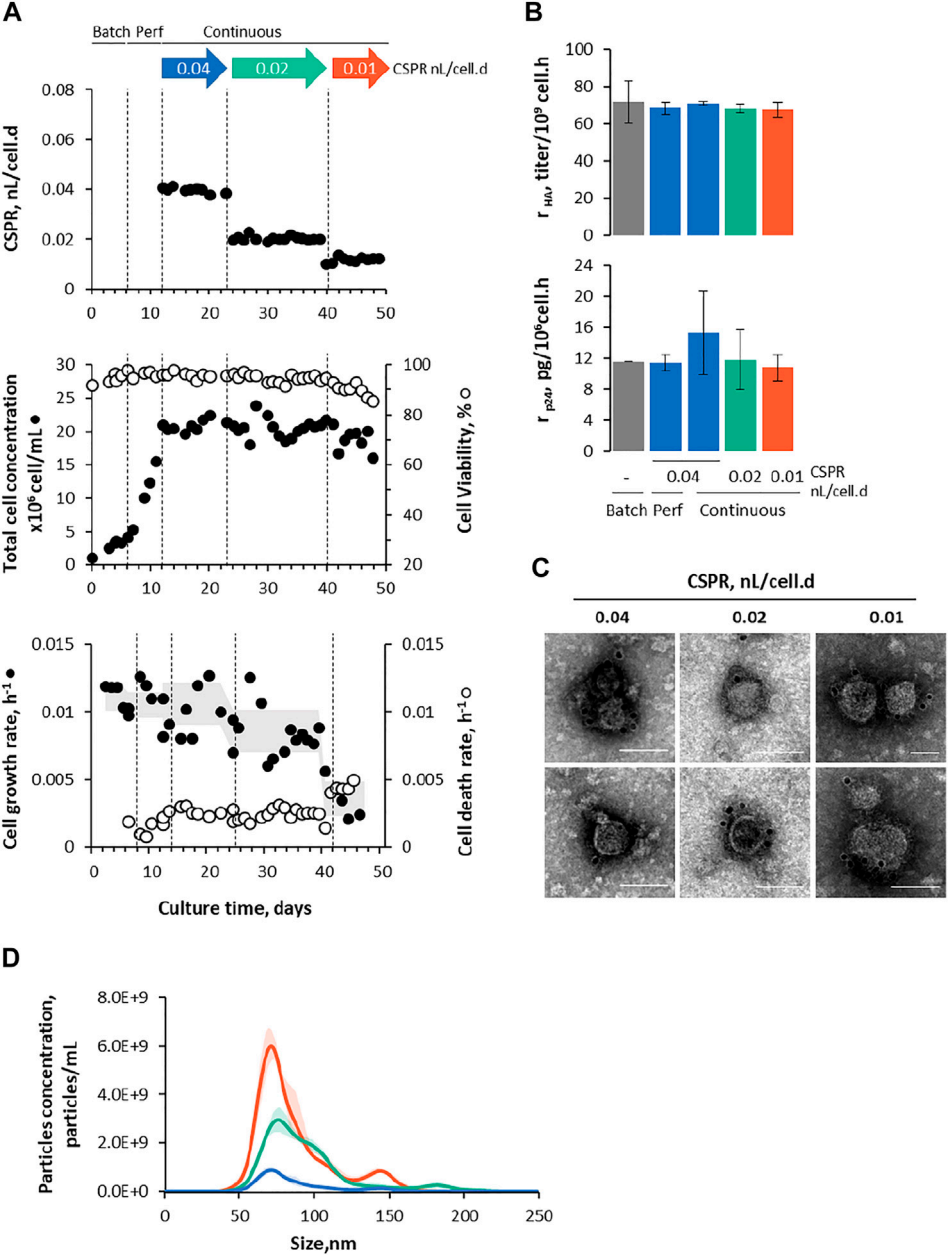
Intensifying continuous Gag-HA VLP expression using ATF. **(A)** The cell-specific perfusion rate (CSPR), cell growth and viability kinetics, and cell growth and death rate. The shadow under the data point is the average cell growth rate variation along culture time. **(B)** Specific HA and p24 production at different modes of operation (batch, perfusion, and continuous) and CSPRs. **(C)** Immunogold staining TEM and **(D)** nanoparticle tracking analysis from purified and concentrated Gag-HA VLP samples. Data in bar graphs are expressed as mean ± SD of one biological replicate using data linearization. One-way ANOVA with post-hoc Tukey’s multiple comparison analysis methods. The mean of each column was compared with the mean of the batch; * adjusted *p*-value < 0.05 was considered statistically significant. Color code: grey represents data with no cell CSPR, blue represents data under CSPR of 0.04 nL/cell.d, green represents data under CSPR of 0.02 nL/cell.d, and orange represents data under CSPR of 0.01 nL/cell.d.

Adapted Sf9 Gag-HA cells were cultured in STB under continuous operation mode using ATF as a cell retention system (Figure 1B), and their growth and protein (p24 and HA) expression kinetics were assessed for up to 36 days under three different CSPR (Figure 3). Cell concentration was maintained at approx. 20 × 10^6^ cell/mL throughout continuous operation mode by manipulating the bleed flow rate (Supplementary Figure S2A).

#### 3.2.1 Gag-HA Virus-Like Particles Production at Cell-Specific Perfusion Rate of 0.04 nL/Cell/D

Cell growth and viability kinetics in ATF were similar to those observed using TFF, with the added benefit of ATF inducing low cell death rates throughout the continuous operation mode (Figure 3A). Likewise, HA and p24 expression seem to be comparable using both cell retention systems (Figure 3B; Supplementary Figures S2B, S2C **)**, and purified Gag-HA VLPs resembled those generated using TFF in terms of size distribution profile and morphology (Figures 3C,D).

#### 3.2.2 Impact of Lowering Cell-Specific Perfusion Rate on Gag-HA Virus-Like Particles Production

Aiming to increase HA and p24 protein titers, a stepwise reduction in CSPR (i.e., 0.04 → 0.02 → 0.01 nL/cell.d) was explored (Figure 3A; Supplementary Figure S2A). Lowering CSPR impacted negatively on the cell growth rate, this being more pronounced at CSPR of 0.01 nL/cell.d (Figure 3A). The cell death rate (k_D_) (assessed by LDH quantification) was rather constant throughout continuous operation mode, the largest variation (i.e., 2-fold increase) was observed when dropping CSPR to 0.01 nL/cell.d, coinciding with a decrease in cell viability to < 90%. Importantly, cell death rates were lower than those achieved using TFF as a cell retention system.

**FIGURE 4:**
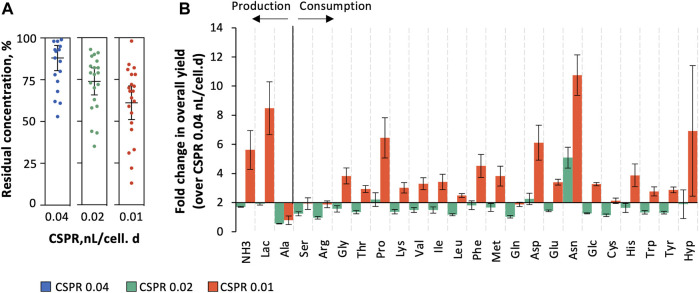
Targeted metabolite profiling assessed by HPLC. **(A)** Residual concentration of amino acids (percent of medium concentration); **(B)** overall yield coefficients for substrate j consumed ( $Y_{\text{X},{\text{S}}_{j}}$ ) or product j formed ( $Y_{\text{X},P_{j}}$ ) per biomass (fold change over CSPR of 0.04 nL/cell.day).

Targeted metabolite profiling (assessed by HPLC) was performed to further understand the impact of operating at different CSPRs on key metabolites and revealed that overall yield coefficients for substrate j consumed ( $Y_{\text{X},{\text{S}}_{j}}$ ) or product j formed ( $Y_{\text{X},P_{j}}$ ) per biomass increases with lowering CSPR, some (e.g., proline, aspartate, asparagine, ammonia, and lactate) registering fold-changes in consumption or production yields of more than four (Figure 4). Notably, none of the amino acids considered in this analysis were depleted (all remained > 13% of the initial concentration in culture medium, on average) nor did they reach growth-impairing concentrations (highest lactate, alanine, and ammonia concentrations were 4.7, 8.4, and 12.9 mM, respectively) (Palomares and Ramirez, 1996; Drugmand et al., 2005) in any of the CSPR tested (Figure 4; Supplementary Table S1).

**FIGURE 5:**
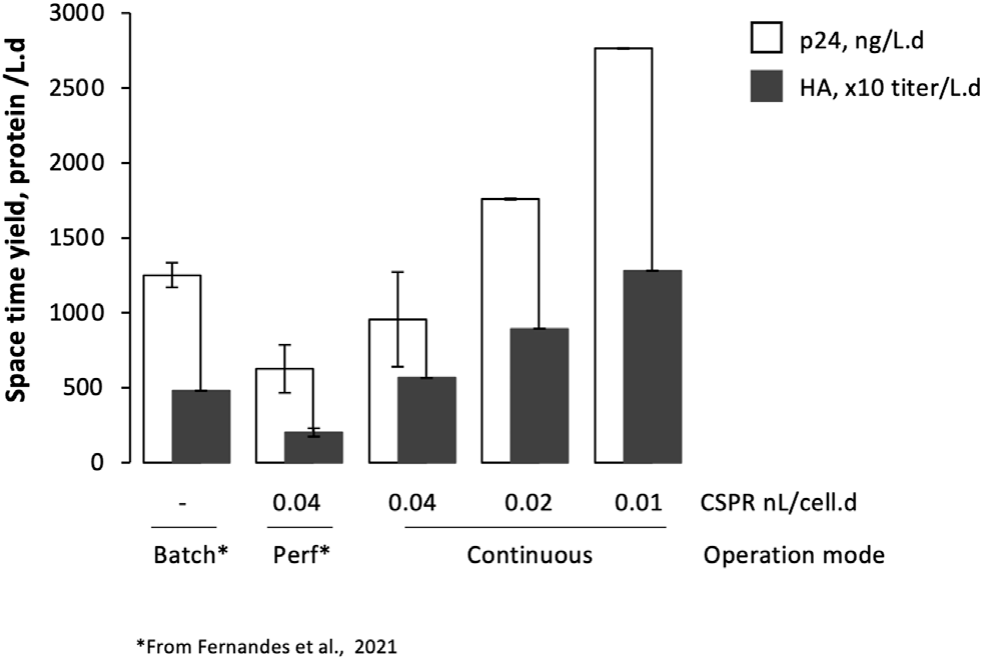
Space-time yield of Gag-HA VLPs produced using stable adapted *Sf9* cells at different bioreactor operation modes and cell-specific perfusion rates. Data from batch and perfusion mode have been reported elsewhere (Fernandes et al., 2021).

HA and p24 proteins could be identified by Western blot (Supplementary Figure S2B); titers increased with the reduction of CSPR, with average product sieving across the hollow fiber membrane > 75% throughout the evaluated CSPR (Supplementary Figure S2C). Specific p24 and HA production rates (r_HA_ and r_p24_, respectively) seem to be similar in all CSPR, and comparable to those obtained in batch and perfusion (Figure 3B). Importantly, space-time yields—defined as the ratio of p24 or HA formed per volume of culture medium consumed per process time—increased with decreasing CSPR (Figure 5), reaching values higher than those observed in previous studies using batch and perfusion operation modes (Fernandes et al., 2021).

Gag-HA VLPs produced in each CSPR were purified, concentrated, and analyzed by TEM and NTA (Figures 3C,D). TEM images confirmed the presence of particles resembling Gag VLPs (in size and morphology) with HA molecules (spikes) displayed on their surface, while NTA data reveals an increase in particle concentration with lowering CSPR.

## 4 Discussion

In this work, a process for continuous Gag-HA VLP production at high cell densities using stable insect cells adapted to low culture temperature was developed. The impact of the cell retention device for perfusion (ATF vs. TFF) and CSPR (0.01 vs. 0.02 vs. 0.04 nL/cell.d) on cell growth and protein expression kinetics was evaluated.

An increase in the cell death rate with culture time using the TFF system was shown, in line with previous reports (Karst et al., 2016; Wang et al., 2017), but this was not shown with ATF. This was more noticeable on day 9 under CSPR of 0.04 nL/cell.d in which differences in the cell death rate between TFF and ATF reached 2-fold. This can be explained by the high shear rates resulting from the peristaltic pump used for cell culture recirculation (Wang et al., 2017). Replacing it with a centrifugal pump that traditionally generates lower shear stress would be an option to mitigate this issue; nevertheless, the hydrodynamic stress one would generate is still above that reported for ATF (80 vs. 20 Pa, for TFF and ATF, respectively) (Karst et al., 2016).

Identifying the minimum CSPR at which continuous operation mode could operate is therefore essential to optimize media utilization thus keeping the cost of goods low (Schulze et al., 2021). The success of decreasing the perfusion rate to minimum values depends on multiple factors, including the relation between specific productivity and perfusion rate, medium formulation and its cost, and finally product stability (Konstantinov et al., 2006). In this study, three CSPRs were evaluated to determine their performance for continuous production of Gag-HA VLPs. The cell growth rate was impaired with lowering CSPR reaching values close to zero at CSPR 0.01 nL/cell.day, with high cell death rates. Targeted metabolite profiling suggests no substrate limitation and/or toxic metabolite(s) accumulation that could justify such cell growth inhibition, similar to what has been described in other studies (Konstantinov et al., 2006). Overall yield coefficients ( $Y_{X,S_{j}}$ or $Y_{X,P_{j}}$ ) increase with reducing CSPR (e.g., Glc, Lac, and Pro), with variations reaching up to 4-fold in some specific metabolites when compared to the initial CSPR (0.04 nL/cell.day). It has been shown that increased glucose consumption and lactate production in cell lines other than those of insect cells is related to cell stress (Keane et al., 2003; Zhan et al., 2020). In addition, proline uptake by mammalian, fungi, and yeast cells is known to play a role in preventing apoptosis and protecting cells from oxidative stress, suggesting that proline works as a universal antioxidant (Chen and Dickman, 2005; Chen et al., 2006; Krishnan et al., 2008). The cell growth rate reducing concomitantly with increased apoptosis, observed in particular at the lowest CSPR, could suggest that cells are under stress. This behavior may result from limitation in key metabolic factors, hence the importance of identifying the lowest limiting concentrations of the nutrients analyzed herein and studying other biological compounds whose limitation or overaccumulation might contribute to cell stress (e.g., proteins, vitamins, or lipids). The development of customized culture media formulations enriched in specific metabolites or culture medium supplementation with key nutrients can prove beneficial in preventing cell stress at lower CSPRs (Sequeira et al., 2018). Decreasing CSPR had no negative impact on p24 and HA expression; in fact, specific protein production rates were rather similar regardless of the CSPR. A similar approach has been successfully applied to antibody production using other cell lines (e.g., CHO and mouse hybridoma cells) (Konstantinov et al., 2006; Schulze et al., 2021). Indeed, by maintaining cell concentration at 20 × 10^6^ cell/mL while decreasing the CSPR, the amount of HA and p24 produced per volume of culture medium consumed per unit of process time (STY) increased by up to 3-fold when compared to previous studies using batch and perfusion operation mode (Fernandes et al., 2021). The presence of particles resembling Gag VLPs (in size and morphology) with HA molecules (spikes) displayed on their surface could be confirmed, with their concentration increasing with reducing CSPR. Working at such low CSPR (i.e., high retention times) requires particles to be stable in culture, as in the case here (Fernandes et al., 2021); for labile proteins and/or protein complexes, the strategy proposed herein may not be suitable and other bioprocessing engineering strategies may be devised such as an end-to-end integrated and continuous process (upstream and downstream) for continuous product harvest and purification (Warikoo et al., 2012; Hiller et al., 2017). Further improvement in specific production rates concomitantly with volumetric titers in the continuous bioprocess established herein for Gag-HA VLP production will require a deeper understanding of stable, adapted insect cell physiology under low CSPR while exploring higher cell densities.

## 5 Conclusion

This study demonstrates the potential of adapted insect cells for continuous production of Gag-HA VLPs at HCD. The bioprocess developed herein allows for an increase in the amount of p24 or HA formed per volume of culture medium consumed per process time when compared to batch and perfusion operation modes. Reducing CSPR enables to optimize expensive media utilization, thus reducing cost without compromising specific protein production rates or particle quality. Integration of upstream and downstream can further intensify the process, making the platform developed herein even more attractive for the continuous production of complex multimeric protein structures such as VLPs.

## Data Availability

The raw data supporting the conclusion of this article will be made available by the authors, without undue reservation.

## Abbreviations

ATF, alternating tangential flow filtration; CSPR, cell-specific perfusion rate; FDA, fluorescein diacetate; Glc, glucose; Gln, glutamine; HA, influenza hemagglutinin; HCD, high cell density; IC-BEVS, insect cell–baculovirus expression vector system; kD, death rate; LDH, lactate dehydrogenase; MW, molecular weight; NTA, nanoparticle tracking analysis; PI, propidium iodide; STY, space-time yield; TEM, transmission electron microscopy; TFF, tangential flow filtration; VLPs, virus-like particles.

## References

[B1] Bielser J.-M. Wolf M. Souquet J. Broly H. Morbidelli M. (2018). Perfusion Mammalian Cell Culture for Recombinant Protein Manufacturing - A Critical Review. Biotechnol. Adv. 36 (4), 1328–1340. 10.1016/j.biotechadv.2018.04.011 29738813

[B2] Chen C. Dickman M. B. (2005). Proline Suppresses Apoptosis in the Fungal Pathogen Colletotrichum Trifolii. Proc. Natl. Acad. Sci. U.S.A. 102 (9), 3459–3464. 10.1073/pnas.0407960102 15699356 PMC552905

[B3] Chen C. Wanduragala S. Becker D. F. Dickman M. B. (2006). Tomato QM-like Protein Protects *Saccharomyces cerevisiae* Cells against Oxidative Stress by Regulating Intracellular Proline Levels. Appl. Environ. Microbiol. 72 (6), 4001–4006. 10.1128/AEM.02428-05 16751508 PMC1489650

[B4] Correia R. Fernandes B. Alves P. M. Carrondo M. J. T. Roldão A. (2020). Improving Influenza HA-Vlps Production in Insect High Five Cells via Adaptive Laboratory Evolution. Vaccines 8 (4), 589. 10.3390/vaccines8040589 PMC7711658 33036359

[B5] Drugmand J.-C. Schneider Y.-J. Agathos S. N. (2005). “Environmental Effects of Lactate on High-Five^TM^ Insect Cell Metabolism,” in Animal Cell Technology Meets Genomics. Editors Gòdia F. Fussenegger M. (Berlin/Heidelberg: Springer-Verlag), 2, 91–94. 10.1007/1-4020-3103-3_14

[B6] Fernandes F. Teixeira A. P. Carinhas N. Carrondo M. J. Alves P. M. (2013). Insect Cells as a Production Platform of Complex Virus-like Particles. Expert Rev. Vaccines 12 (2), 225–236. 10.1586/erv.12.153 23414412

[B7] Fernandes B. Vidigal J. Correia R. Carrondo M. J. T. Alves P. M. Teixeira A. P. (2020). Adaptive Laboratory Evolution of Stable Insect Cell Lines for Improved HIV-Gag VLPs Production. J. Biotechnol. 307, 139–147. 10.1016/j.jbiotec.2019.10.004 31697977

[B8] Fernandes B. Correia R. Sousa M. Carrondo M. J. T. Alves P. M. Roldão A. (2021). Integrating High Cell Density Cultures with Adapted Laboratory Evolution for Improved Gag‐HA Virus‐like Particles Production in Stable Insect Cell Lines. Biotechnol. Bioeng. 118 (7), 2536–2547. 10.1002/bit.27766 33764532

[B10] Hiller G. W. Ovalle A. M. Gagnon M. P. Curran M. L. Wang W. (2017). Cell-controlled Hybrid Perfusion Fed-Batch CHO Cell Process Provides Significant Productivity Improvement over Conventional Fed-Batch Cultures: Hybrid Perfusion Fed-Batch CHO Culture. Biotechnol. Bioeng. 114 (7), 1438–1447. 10.1002/bit.26259 28128436

[B11] Karst D. J. Serra E. Villiger T. K. Soos M. Morbidelli M. (2016). Characterization and Comparison of ATF and TFF in Stirred Bioreactors for Continuous Mammalian Cell Culture Processes. Biochem. Eng. J. 110, 17–26. 10.1016/j.bej.2016.02.003

[B12] Keane J. T. Ryan D. Gray P. P. (2003). Effect of Shear Stress on Expression of a Recombinant Protein by Chinese Hamster Ovary Cells. Biotechnol. Bioeng. 81 (2), 211–220. 10.1002/bit.10472 12451557

[B13] Konstantinov K. Goudar C. Ng M. Meneses R. Thrift J. Chuppa S. (2006). “The "Push-To-Low" Approach for Optimization of High-Density Perfusion Cultures of Animal Cells,” in Cell Culture Engineering. Editor Hu W.-S. (Berlin/Heidelberg: Springer), 101, 75–98. 10.1007/10_016 16989258

[B14] Krammer F. Grabherr R. (2010). Alternative Influenza Vaccines Made by Insect Cells. Trends Mol. Med. 16 (7), 313–320. 10.1016/j.molmed.2010.05.002 20570562

[B15] Krammer F. Schinko T. Palmberger D. Tauer C. Messner P. Grabherr R. (2010). Trichoplusia Ni Cells (High FiveTM) Are Highly Efficient for the Production of Influenza A Virus-like Particles: A Comparison of Two Insect Cell Lines as Production Platforms for Influenza Vaccines. Mol. Biotechnol. 45 (3), 226–234. 10.1007/s12033-010-9268-3 20300881 PMC4388404

[B16] Krishnan N. Dickman M. B. Becker D. F. (2008). Proline Modulates the Intracellular Redox Environment and Protects Mammalian Cells against Oxidative Stress. Free Radic. Biol. Med. 44 (4), 671–681. 10.1016/j.freeradbiomed.2007.10.054 18036351 PMC2268104

[B17] Kuwahara M. Konishi E. (2010). Evaluation of Extracellular Subviral Particles of Dengue Virus Type 2 and Japanese Encephalitis Virus Produced by Spodoptera Frugiperda Cells for Use as Vaccine and Diagnostic Antigens. Clin. Vaccine Immunol. 17 (10), 1560–1566. 10.1128/CVI.00087-10 20668137 PMC2952984

[B18] Lin H. Leighty R. W. Godfrey S. Wang S. B. (2017). Principles and Approach to Developing Mammalian Cell Culture Media for High Cell Density Perfusion Process Leveraging Established Fed-Batch Media. Biotechnol. Prog. 33 (4), 891–901. 10.1002/btpr.2472 28371394

[B19] Lynch A. G. Tanzer F. Fraser M. J. Shephard E. G. Williamson A.-L. Rybicki E. P. (2010). Use of the piggyBac Transposon to Create HIV-1 Gag Transgenic Insect Cell Lines for Continuous VLP Production. BMC Biotechnol. 10 (1), 30. 10.1186/1472-6750-10-30 20356379 PMC2853493

[B20] Mahase E. (2021). Covid-19: Novavax Vaccine Efficacy is 86% against UK Variant and 60% against South African Variant. BMJ 372, n296. 10.1136/bmj.n296 33526412

[B21] Metz S. W. Gardner J. Geertsema C. Le T. T. Goh L. Vlak J. M. (2013). Effective Chikungunya Virus-like Particle Vaccine Produced in Insect Cells. PLoS Negl. Trop. Dis. 7 (3), e2124. 10.1371/journal.pntd.0002124 23516657 PMC3597470

[B22] Mortola E. Roy P. (2004). Efficient Assembly and Release of SARS Coronavirus-like Particles by a Heterologous Expression System. FEBS Lett. 576 (1–2), 174–178. 10.1016/j.febslet.2004.09.009 15474033 PMC7126153

[B23] Nikolay A. Grooth J. Genzel Y. Wood J. A. Reichl U. (2020). Virus Harvesting in Perfusion Culture: Choosing the Right Type of Hollow Fiber Membrane. Biotechnol. Bioeng. 117 (10), 3040–3052. 10.1002/bit.27470 32568408

[B38] Nooraei S. Bahrulolum H. Hoseini Z. S. Katalani C. Hajizade A. Easton A. J. (2021). Virus-Like Particles: Preparation, Immunogenicity and Their Roles as Nanovaccines and Drug Nanocarriers. J. Nanobiotechnol. 19, 59. 10.1186/s12951-021-00806-7 PMC7905985 33632278

[B24] Palomares L. A. Ramirez O. T. (1996). The Effect of Dissolved Oxygen Tension and the Utility of Oxygen Uptake Rate in Insect Cell Culture. Cytotechnology 22 (1–3), 225–237. 10.1007/BF00353943 22358933

[B25] Pushko P. Tumpey T. Bu F. Knell J. Robinson R. Smith G. (2005). Influenza Virus-like Particles Comprised of the HA, NA, and M1 Proteins of H9N2 Influenza Virus Induce Protective Immune Responses in BALB/c Mice. Vaccine 23 (50), 5751–5759. 10.1016/j.vaccine.2005.07.098 16143432

[B26] Radhakrishnan D. Wells E. A. Robinson A. S. (2018). Strategies to Enhance Productivity and Modify Product Quality in Therapeutic Proteins. Curr. Opin. Chem. Eng. 22, 81–88. 10.1016/j.coche.2018.09.005

[B27] Roldão A. Mellado M. C. M. Castilho L. R. Carrondo M. J. Alves P. M. (2010). Virus-like Particles in Vaccine Development. Expert Rev. Vaccines 9 (10), 1149–1176. 10.1586/erv.10.115 20923267

[B28] Schulze M. Lemke J. Pollard D. Wijffels R. H. Matuszczyk J. Martens D. E. (2021). Automation of High CHO Cell Density Seed Intensification via Online Control of the Cell Specific Perfusion Rate and its Impact on the N-Stage Inoculum Quality. J. Biotechnol. 335, 65–75. 10.1016/j.jbiotec.2021.06.011 34090946

[B29] Sequeira D. P. Correia R. Carrondo M. J. T. Roldão A. Teixeira A. P. Alves P. M. (2018). Combining Stable Insect Cell Lines with Baculovirus-Mediated Expression for Multi-HA Influenza VLP Production. Vaccine 36 (22), 3112–3123. 10.1016/j.vaccine.2017.02.043 28291648

[B30] Sun Y. Carrion R. Ye L. Wen Z. Ro Y.-T. Brasky K. (2009). Protection against Lethal Challenge by Ebola Virus-like Particles Produced in Insect Cells. Virology 383 (1), 12–21. 10.1016/j.virol.2008.09.020 18986663 PMC2657000

[B31] Tapia F. Vázquez-Ramírez D. Genzel Y. Reichl U. (2016). Bioreactors for High Cell Density and Continuous Multi-Stage Cultivations: Options for Process Intensification in Cell Culture-Based Viral Vaccine Production. Appl. Microbiol. Biotechnol. 100 (5), 2121–2132. 10.1007/s00253-015-7267-9 26758296 PMC4756030

[B32] Tretyakova I. Hidajat R. Hamilton G. Horn N. Nickols B. Prather R. O. (2016). Preparation of Quadri-Subtype Influenza Virus-like Particles Using Bovine Immunodeficiency Virus Gag Protein. Virology 487, 163–171. 10.1016/j.virol.2015.10.007 26529299 PMC4679414

[B33] Voisard D. Meuwly F. Ruffieux P.-A. Baer G. Kadouri A. (2003). Potential of Cell Retention Techniques for Large-Scale High-Density Perfusion Culture of Suspended Mammalian Cells. Biotechnol. Bioeng. 82 (7), 751–765. 10.1002/bit.10629 12701141

[B35] Wang S. Godfrey S. Ravikrishnan J. Lin H. Vogel J. Coffman J. (2017). Shear Contributions to Cell Culture Performance and Product Recovery in ATF and TFF Perfusion Systems. J. Biotechnol. 246, 52–60. 10.1016/j.jbiotec.2017.01.020 28159614

[B36] Warikoo V. Godawat R. Brower K. Jain S. Cummings D. Simons E. (2012). Integrated Continuous Production of Recombinant Therapeutic Proteins. Biotechnol. Bioeng. 109 (12), 3018–3029. 10.1002/bit.24584 22729761

[B37] Zhan C. Bidkhori G. Schwarz H. Malm M. Mebrahtu A. Field R. (2020). Low Shear Stress Increases Recombinant Protein Production and High Shear Stress Increases Apoptosis in Human Cells. IScience 23 (11), 101653. 10.1016/j.isci.2020.101653 33145483 PMC7593556

