# Supplementary material for: Intensifying Continuous Production of Gag-HA VLPs at High Cell Density Using Stable Insect Cells Adapted to Low Culture Temperature
Source: Front Bioeng Biotechnol. 2022 Jun 29;10:917746. doi: 10.3389/fbioe.2022.917746 (PMC9277389; doi:10.3389/fbioe.2022.917746)
Supplement: Supplementary file 1 [file DataSheet1.pdf]

Supplementary Information

Mathematical equations for estimation of reaction rates

Cell specific growth rate, μ, is given by:

$$\mu\ (h^{-1}) = \frac{1}{X} \cdot \frac{dx}{dt} \qquad \text{(Eq.1)}$$

where X is the concentration of viable cells (cell/mL) and t is the culture time (h).

The yield coefficients, $Y_{i,j}$ (mass of j formed or consumed *per* mass of i formed or consumed), were estimated using the formulation of) (Nielsen, Villadsen, & Lidén, 2003) (Nielsen et al., 2003). Important, the yield coefficients determined in batch experiments are the overall yield coefficients and not $Y_{i,j}$ (associated with the specific growth rate and thus time dependent) or $Y_{i,j}^{True}$ (mass of j formed or consumed *per* mass of i consumed only for the conversion, not accounting for the maintenance coefficient m_S expressed as a substrate demand to maintain cell viability per unit of cells per time as defined in (Pirt, 1965)).

The overall yield coefficients for mass of product j formed per biomass, Y_{X,P_j} , and for mass of substrate j consumed per biomass, $Y_{\mathrm{X},\mathrm{S}_j}$, are defined as follows:

$$Y_{\mathrm{X},P_{p24}}(ng.cell^{-1}) = \Delta P_{p24}/\Delta \mathrm{X} \quad , \quad 0 < t < t_\alpha \qquad \text{(Eq.2)}$$

$$Y_{\mathrm{X},P_{HA}}(HA\ titer.cell^{-1}) = \Delta P_{HA}/\Delta \mathrm{X} \quad , \quad 0 < t < t_\alpha \qquad \text{(Eq.3)}$$

$$Y_{\mathrm{X},\mathrm{S}_{Glc\ or\ Gln}}(mol.cell^{-1}) = \Delta S_{Glc\ or\ Gln}/\Delta \mathrm{X} \quad , \quad 0 < t < t_\beta \qquad \text{(Eq.4)}$$

where P is the concentration of product j formed, either p24 (ng/mL) or HA (HA titer/mL) protein, S is the concentration of substrate j consumed, either glucose (Glc, mM) or glutamine (Gln, mM), t_α is the culture time at which maximum cell concentration is achieved for a cell

viability above 90 %, and t_{β} is the culture time at which linear cell growth is no longer observed.

The mass balance equation on substrate for batch, perfusion and continuous processes is described by:

$$\frac{d(S_j.V)}{dt} = -r_{S_j}XV + F_{in}S_j^{in} - (F_{out}\,S_j^{out} + F_{bleed}S_j^{bleed}) \quad \text{(Eq.5)}$$

where V (L) is the bioreactor volume, r_{S_j} (mol/cell.h) is the specific rate of substrate j (Glc or Gln) consumed, F_{in} (L/h) is the flow in of fresh medium, F_{out} (L/h) is the flow out of spent medium, F_{bleed} (L/h) is the flow of cells bleed of the bioreactor (applicable to continuous mode), S_j^{in} (mM) is the concentration of substrate j in the flow in, and S_j^{out} (mM) is the concentration of substrate j in the flow out, and S_j^{bleed} (mM) is the concentration of substrate j in the flow of cells bleed. For batch, $F_{in} = F_{out} = 0$, which by rearranging Eqs. 1 and 5, gives r_{S_j} as follows:

$$r_{S_{Glc\ or\ Gln}}\ (mol.cell^{-1}.h^{-1}) = \frac{1}{Y_{X,S_j}} \cdot \mu \quad \text{(Eq.6)}$$

For perfusion, $F_{in} = F_{out} \neq 0$, which by rearranging Eq. 5, gives r_{S_j} as follows:

$$r_{S_{Glc\ or\ Gln}}\ (mol.cell^{-1}.h^{-1}) = \frac{1}{X} \cdot \left[\frac{\Delta S_j}{\Delta t} + \frac{F}{V}\left(S_j^{in} - S_j^{out\ med}\right)\right] \quad \text{(Eq.7)}$$

For continuous, $F_{in} = F_{out} + F_{bleed} \neq 0$, which by rearranging Eq. 5, gives r_{S_j} as follows:

$$r_{S_{Glc\ or\ Gln}}\ (mol.cell^{-1}.h^{-1}) = \frac{1}{X} \cdot \left[\frac{\Delta S_j}{\Delta t} + \frac{F}{V}\left(S_j^{in} - (S_j^{out} + S_j^{bleed}\right)\right] \quad \text{(Eq.7)}$$

As the p24 and HA protein are a mixed-growth associated product (products appearing during exponential phase and also slow growth and stationary phases) (Gutiérrez-Granados et al.,

2015; Venereo-Sanchez et al., 2017), the specific rate of p24 and HA protein formation, $r_{P_{p24}}$ and $r_{P_{HA}}$, respectively is given by:

$$r_{P_{p24}}\ (pg.cell^{-1}.h^{-1}) = \Delta P_{p24}\ /\int_0^{t_\alpha} X\ dt \qquad \text{(Eq.8)}$$

$$r_{P_{HA}}\ (HA\ titer.cell^{-1}.h^{-1}) = \Delta P_{HA}\ /\int_0^{t_\alpha} X\ dt \qquad \text{(Eq.9)}$$

The cell dilution rate (CDR) equation is described by:

$$\mathrm{CDR}\ (\mathrm{mL}.h^{-1}) = \mu\ \times \mathrm{V} \qquad \text{(Eq.10)}$$

where μ (h^{-1}) is growth rate and V (mL) is the bioreactor volume.

Cell death rate (k_D) by lactate dehydrogenase (LDH) was calculated using the following equations:

$$\mathrm{LDH/cell}\ = \frac{[LDH]_{triton\ X-100} - [LDH]_{untreated}}{X_{triton\ X-100} - X_{untreated}} \qquad \text{(Eq.11)}$$

$$q_{\mathrm{LDH}}(\mathrm{U}.10^{-6}\mathrm{cells}.h^{-1}) = \frac{1}{X} \times ([LDH] \times\ PR) \qquad \text{(Eq.12)}$$

$$k_D\ (h^{-1}) = \frac{q_{LDH}}{LDH/cell} \qquad \text{(Eq.13)}$$

where q_{LDH} is the cell specific LDH productivity and PR is the perfusion rate (L/h). Cell lysis was induced by adding to cell culture samples 10% of Triton X-100 (Sigma) (Wang et al., 2017).

Space time yield (STY) was calculated using the following equation:

$$STY\ (ng.L^{-1}.d^{-1}) = \frac{protein\ (ng)}{media\ spent\ (L) \times culture\ time\ (days)}$$

Supplementary Figures

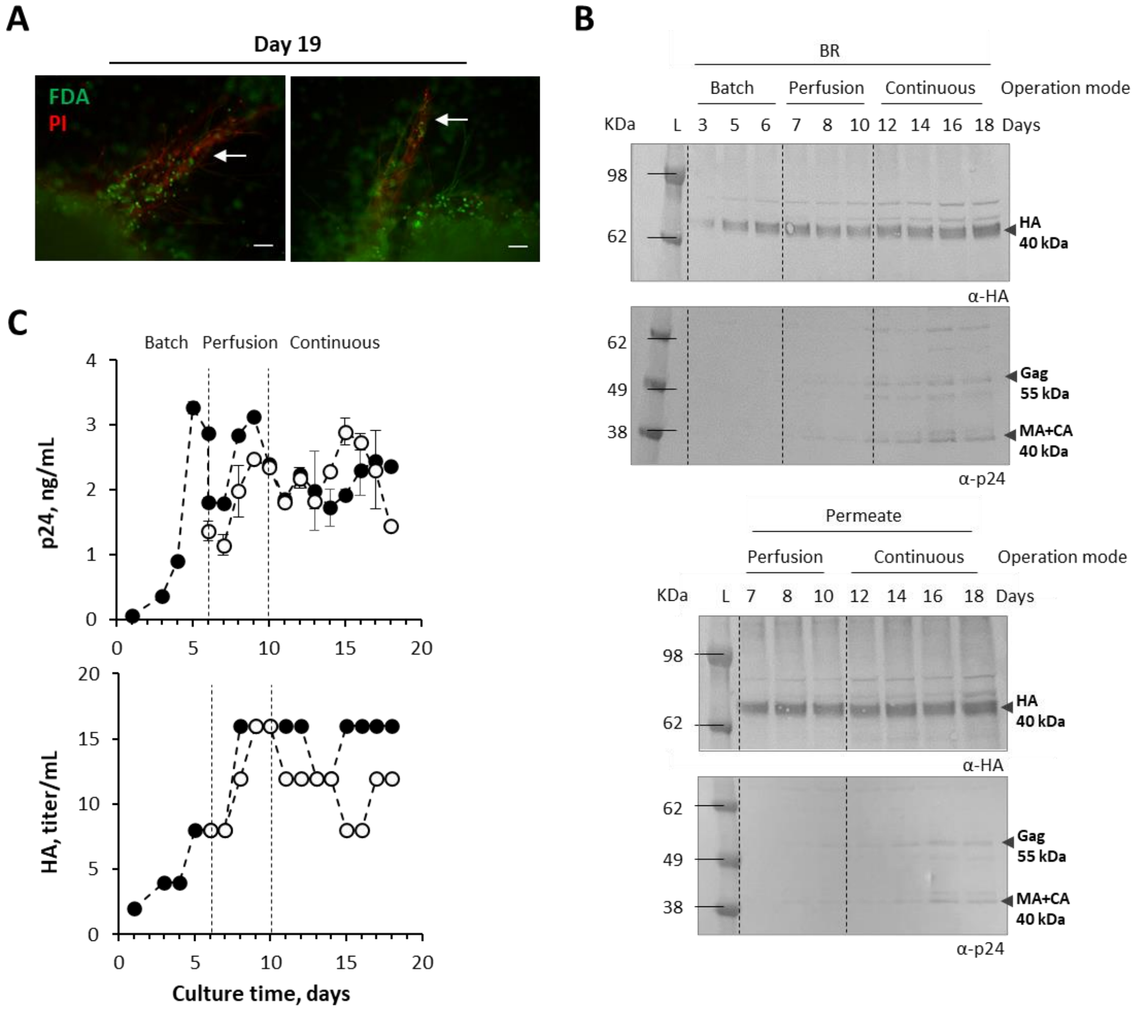

Figure S1. Production of Gag-HA VLPs in continuous operation mode using TFF (A) Fluorescence microcopy of cells at day 19 of culture. Live cells were stained with fluorescein diacetate (FDA, green) and dead cells/double-stranded DNA with propidium iodide (PI, red). (B) Identification of p24 and HA protein samples collected from the bioreactor and the permeate bottles by western blot. L denote the SeeBlue™ Plus2 Pre-stained Protein standard. MA denote for matrix protein and CA denote for capsid protein of Gag. (C) HA and p24 concentration in the bioreactor (●) and in the permeate bottles (○) along culture time.

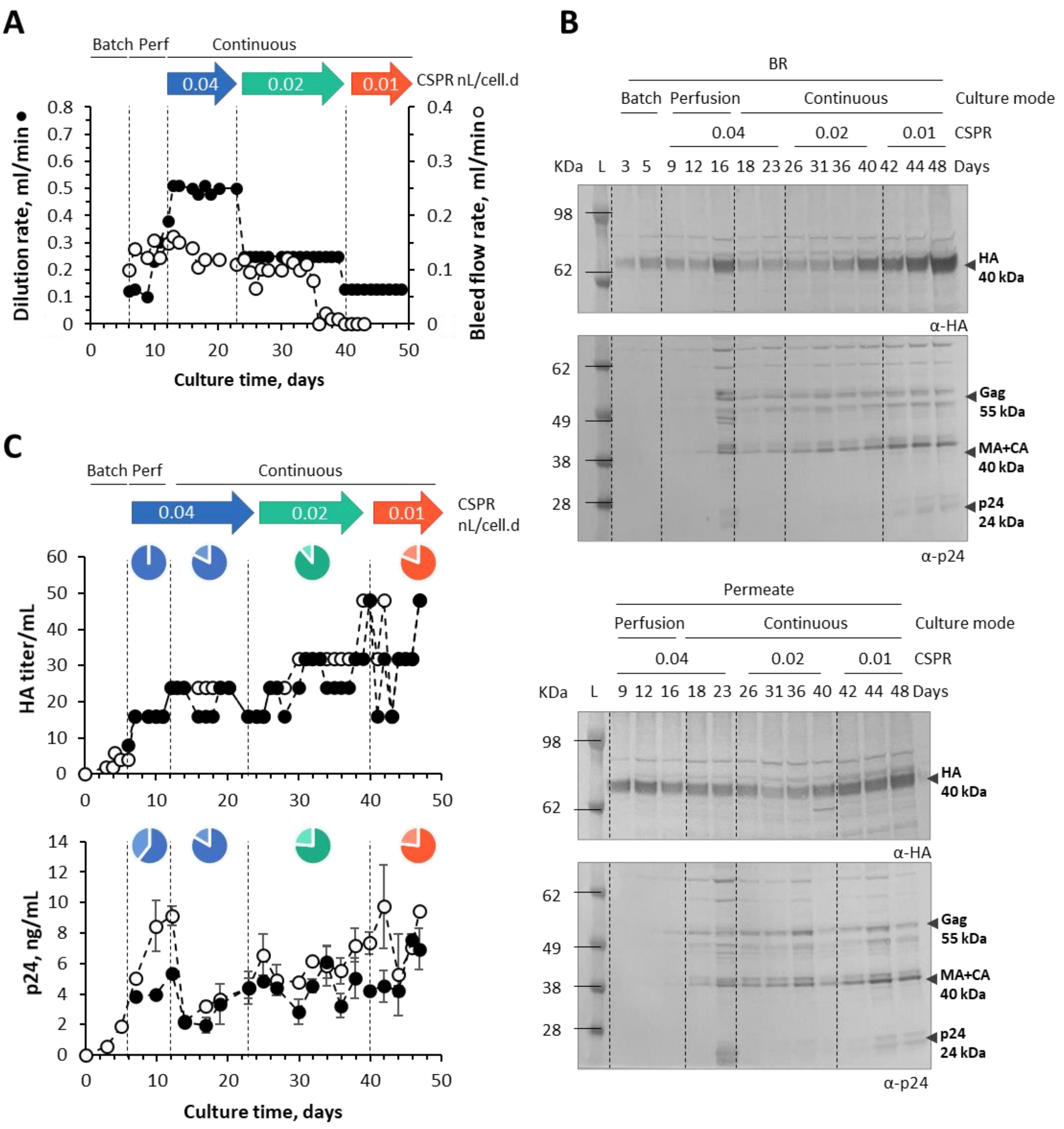

Figure S2. Intensifying continuous Gag-HA VLPs expression using ATF. (A) Dilution rate (●) and bleed flow rate (○). (B) Identification of p24 and HA protein samples collected from the bioreactor and the permeate bottles by western blot. L denote the SeeBlue™ Plus2 Pre-stained Protein standard. MA denote for matrix protein and CA denote for capsid protein of Gag. (C) HA and p24 concentration in the bioreactor (●) and in the permeate bottles (○) along culture time. Pie charts represent the average product sieving during the different phases (operations modes and CSPRs), dark color represent the average percentage of protein

permeated and the light color represent the average percentage of protein inside the bioreactor in that specific phase of the culture.

Supplementary Table

Table 1. Residual concentration of amino acids in the medium and in the bioreactor using different cell specific perfusion rate (CSPR) for the continuous production of Gag-HA VLPs.

Amino acids	Culture medium	Bioreactor CSPR nL/cell.d		
		0.04	0.02	0.01
**NH3**	2.5	5.6	8.3	12.9
**Ala**	2.9	8.4	6.1	4.7
**Lac**	0.2	1.2	2.3	4.7
**Hyp**	5.8	6.4	5.9	5.7
**His**	1.5	1.4	1.2	1.0
**Asn**	10.7	11.6	10.0	9.0
**Ser**	3.8	2.0	1.3	0.5
**Gln**	12.4	8.4	7.3	6.1
**Arg**	4.2	3.3	3.1	2.7
**Gly**	4.0	3.9	3.3	2.8
**Asp**	11.8	12.4	10.6	9.2
**Glu**	13.7	12.7	11.4	9.9
**Thr**	2.8	2.4	2.0	1.6
**Pro**	5.1	5.1	4.1	3.1
**Cys2**	0.4	0.2	0.2	0.1
**Lys**	5.2	4.8	4.1	3.5
**Tyr**	1.3	1.1	0.8	0.6
**Met**	6.5	6.6	5.8	5.3
**Val**	5.7	5.4	4.7	4.0
**Ile**	6.4	6.3	5.5	5.0
**Leu**	2.7	1.7	1.2	0.6
**Phe**	6.4	6.6	5.8	5.2
**Trp**	0.7	0.6	0.5	0.4
**Glucose**	60.9	45.3	35.1	20.1
**Culture time, days**	-	23	40	48
